# Supplementary material for: Long-term respiratory follow-up of ICU hospitalized COVID-19 patients: Prospective cohort study
Source: PLoS One. 2023 Jan 20;18(1):e0280567. doi: 10.1371/journal.pone.0280567 (PMC9858876; doi:10.1371/journal.pone.0280567)
Supplement: S1 Appendix — Classification of computed tomography (CT) lung lesions. ML prediction model: training and validation. Results. Implementations notes. References. (DOCX) [file pone.0280567.s001.docx]

**Supporting Information S1 Appendix**

**Long-term respiratory follow-up of ICU hospitalized COVID-19 patients: prospective cohort study**

Carlos Roberto Ribeiro Carvalho1, Celina Almeida Lamas1, Rodrigo Caruso Chate2, João Marcos Salge1, Marcio Valente Yamada Sawamura2, André L. P. de Albuquerque1, Carlos Toufen Junior1, Daniel Mario Lima3, Michelle Louvaes Garcia1, Paula Gobi Scudeller1, Cesar Higa Nomura2, Marco Antonio Gutierrez3, Bruno Guedes Baldi1, HCFMUSP Covid-19 Study Group*

1 Pulmonary Division, Heart Institute (InCor), Hospital das Clínicas, Faculdade de Medicina, Universidade de São Paulo (HCFMUSP), Sao Paulo, SP, Brazil.

2 Radiology Institute (InRad), Hospital das Clínicas, Faculdade de Medicina, Universidade de São Paulo (HCFMUSP), Sao Paulo, SP, Brazil.

3 Informatics Division, Heart Institute (InCor), Hospital das Clínicas, Faculdade de Medicina, Universidade de São Paulo (HCFMUSP), Sao Paulo, SP, Brazil.

*The complete membership of the author group can be found in the Acknowledgments.

**S1 Appendix**

**Supplemental Machine Learning Model (ML) Information’s**

**Classification of computed tomography (CT) lung lesions**

The group of 175 patients that had lung with COVID-19 findings were sub stratified into two further groups according to lesion severity: without fibrotic-like changes (with presence of ground-glass opacities and reticulations) (N=89) and with fibrotic-like changes (with presence of traction bronchiectasis and architectural distortions) (N=86). Based on clinical parameters observed in these patients, we developed a ML model to predict the lesion severity after six to twelve months from the admission of the ICU.

**ML prediction model: training and validation**

Predicting the lesion severity was posed as a classification task, and we assessed the efficacy of competing ML models for this task. Specifically, Random Forest (1) and XGBoost (2) (extreme gradient boost), two well-known ensemble models, usually superior to single models in terms of generalizability and robustness. (3) The random forest, a bagging type of ensemble, consists of multiple independent decision trees, each of which is trained using a random subset of features and a random subset of samples drawn with replacement, and hence enjoys reduced variance of the ensemble, avoiding overfitting. In contrast, XGBoost makes use of a type of gradient boosting, where multiple decision tree models are trained in succession, each tending to improve performance.

The dataset with 175 patients was divided into balanced training and validation sets, according to lesion severity (Table 1). A three-fold cross-validation strategy was adopted for the training and validation sets. The hyperparameters in Random Forest were adjusted to 100 trees, 2 minimum samples required to split an internal node, and 1 minimum number of samples, required being at a leaf node. XGBoost parameters were adjusted to L2 and L1 regularisation terms, 0.7217 and 0.0201, respectively, subsample ratio of the training instances 0.0340, and subsample ratio of columns 0.7247. The variables collected at baseline with p<0.05 between two categories of CT lesion severity (without fibrotic-like changes (N=89) and with fibrotic-like changes (N=86)) were used as input variable into the ML models: sex (%), ICU length of stay (days), tracheostomy (%), duration of IMV (days) and the use of vasoactive drug (%).

| Table 1. Distribution of patients into balanced training and validation sets. | | | |
| --- | --- | --- | --- |
| Lesion severity classification | **Number of patients** | | |
|  | **Total** | **Train set** | **Validation Set** |
| Without Fibrotic-like Changes | 89 | 57 | 32 |
| With Fibrotic-like Changes | 86 | 59 | 27 |

**Results**

The observed performance metrics of the ML prediction models are described in Table 2. In terms of the area under the curve (AUC), the XGBoost model showed a better performance (AUC = 0.83 ± 0.01) than Random Forest (AUC = 0.78 ± 0.01). A two-sample t-Test showed a p-value = 0.008 (significance level 0.05) and we reject the null hypothesis. In view of this, we choose the XGBoost ML to be used in the study.

| Table 2. Performance metrics of the ML prediction models. | | | | | | | |
| --- | --- | --- | --- | --- | --- | --- | --- |
| ML  Prediction  Model | **3-fold** | **Sensitivity** | **Specificity** | **F1-score** | **Positive Predictive Rate** | **Accuracy** | **AUC** |
| Random  Forest | 1 | 0.74 | 0.81 | 0.74 | 0.75 | 0.74 | 0.77 |
|  | 2 | 0.74 | 0.81 | 0.74 | 0.75 | 0.74 | 0.78 |
|  | 3 | 0.77 | 0.82 | 0.76 | 0.75 | 0.73 | 0.79 |
|  | Mean ± SD | 0.75 ± 0.01 | 0.82 ± 0.01 | 0.75 ± 0.02 | 0.75 ± 0.01 | 0.74 ± 0.00 | **0.78 ± 0.01** |
|  | 95% CI | 0.74 – 0.76 | 0.81 – 0.83 | 0.73 – 0.77 | 0.74 – 0.76 | 0.74 – 0.74 | 0.77 – 0.79 |
| XGBoost | 1 | 0.76 | 0.78 | 0.76 | 0.76 | 0.76 | 0.82 |
|  | 2 | 0.78 | 0.79 | 0.78 | 0.78 | 0.78 | 0.83 |
|  | 3 | 0.79 | 0.79 | 0.79 | 0.8 | 0.8 | 0.83 |
|  | Mean ± SD | 0.78 ± 0.02 | 0.79 ± 0.01 | 0.78 ± 0.02 | 0.78 ± 0.02 | 0.78 ± 0.02 | **0.83 ± 0.01** |
|  | 95% CI | 0.76 – 0.79 | 0.78 – 0.8 | 0.76 – 0.8 | 0.76 – 0.8 | 0.76 – 0.8 | 0.82 – 0.83 |
| *Abbreviations: CI, confidence interval; SD, standard deviation.* | | | | | | | |

**Implementations notes**

Computer coding was carried out in Python programming language version 3.8 and Random Forest and XGBoost algorithms from the Scikit Learn v.0.24 library.

**References**

1. Palimkar P, Shaw RN, Ghosh A. Machine Learning Technique to Prognosis Diabetes Disease: Random Forest Classifier Approach BT - Advanced Computing and Intelligent Technologies. In: Bianchini M, Piuri V, Das S, Shaw RN, editors. Singapore: Springer Singapore; 2022. p. 219–44.

2. Alsahaf A, Petkov N, Shenoy V, Azzopardi G. A framework for feature selection through boosting. Expert Syst Appl [Internet]. 2022;187:115895. Available from: https://www.sciencedirect.com/science/article/pii/S0957417421012513

3. Sagi O, Rokach L. Ensemble learning: A survey. WIREs Data Min Knowl Discov [Internet]. 2018 Jul 1;8(4):e1249. Available from: https://doi.org/10.1002/widm.1249
