# Supplementary material for: Long-term respiratory follow-up of ICU hospitalized COVID-19 patients: Prospective cohort study
Source: PLoS One. 2023 Jan 20;18(1):e0280567. doi: 10.1371/journal.pone.0280567 (PMC9858876; doi:10.1371/journal.pone.0280567)
Supplement: S3 Table — Values are presented as median [IQR, n] or % (n/N) or mean ± SD (n). Abbreviations: BMI, body mass index; COPD, chronic obstructive pulmonary disease; CRP, c-reactive protein; d, days; FIO2, inspired fraction of oxygen; ICU, intensive care unit. IMV, invasive mechanical ventilation; PaO2, arterial partial pressure of oxygen; PEEP, positive end-expiratory pressure; PFTs, pulmonary function tests; SAPS3, Simplified Acute Physiology Score 3; SD, standard deviation; VAD, vasoactive drugs; yr, years. (DOCX) [file pone.0280567.s004.docx]

**Supporting Information S3 Table**

**Long-term respiratory follow-up of ICU hospitalized COVID-19 patients: prospective cohort study**

Carlos Roberto Ribeiro Carvalho1, Celina Almeida Lamas1, Rodrigo Caruso Chate2, João Marcos Salge1, Marcio Valente Yamada Sawamura2, André L. P. de Albuquerque1, Carlos Toufen Junior1, Daniel Mario Lima3, Michelle Louvaes Garcia1, Paula Gobi Scudeller1, Cesar Higa Nomura2, Marco Antonio Gutierrez3, Bruno Guedes Baldi1, HCFMUSP Covid-19 Study Group*

1 Pulmonary Division, Heart Institute (InCor), Hospital das Clínicas, Faculdade de Medicina, Universidade de São Paulo (HCFMUSP), Sao Paulo, SP, Brazil.

2 Radiology Institute (InRad), Hospital das Clínicas, Faculdade de Medicina, Universidade de São Paulo (HCFMUSP), Sao Paulo, SP, Brazil.

3 Informatics Division, Heart Institute (InCor), Hospital das Clínicas, Faculdade de Medicina, Universidade de São Paulo (HCFMUSP), Sao Paulo, SP, Brazil.

*The complete membership of the author group can be found in the Acknowledgments.

**S3 Table.**

| S3 Table. Demographic and clinical characteristics of patients with signs of pulmonary involvement stratified by completion of lung function examination. | | | |
| --- | --- | --- | --- |
|  | **Patients with PFTs results (N=214)** | **Patients without PFTs results**  **(N=29)** | **p-value** |
| Demographics |  |  |  |
| Age, mean ± SD (n) - yr | 56.1 ± 13.1 (n=214) | 57.7 ± 14.3 (n=29) | 0.536 |
| Male, % (n/N) | 50 (107/214) | 27.6 (8/29) | 0.029 |
| BMI, median (IQR, n) - kg/m^2^ | 32 (28.2 - 36.5, n=214) | 30.3 (28.5 - 34.6, n=29) | 0.155 |
| Comorbidities |  |  |  |
| Chronic Kidney Disease, % (n/N) | 7.5 (16/214) | 7 (2/29) | 1 |
| Diabetes, % (n/N) | 40.2 (86/214) | 58.6 (17/29) | 0.072 |
| COPD, % (n/N) | 9.3 (20/214) | 0 (0/29) | 0.142 |
| Hypertension, % (n/N) | 58.9 (126/214) | 69 (20/29) | 0.321 |
| Smoke History, % (n/N) | 41.6 (89/214) | 37.9 (11/29) | 0.841 |
| Characteristics in ICU |  |  |  |
| ICU lenght of stay, median (IQR, n) - d | 11 (6 - 20, n=214) | 12 (8 - 16, n=29) | 0.340 |
| SAPS 3 at admission, median (IQR, n) | 56 (46 - 69, n=207) | 62 (54 - 68, n=29) | 0.032 |
| D Dimer 72h, median (IQR, n) - ng/ml | 1598 (896.7 - 4396.5, n=202) | 1749 (877.5 - 4915.7, n=28) | 0.430 |
| CRP 72h, median (IQR, n) - mg/l | 155.7 (79.4 - 243.5, n=205) | 84.2 (53.2 - 241.7, n=29) | 0.096 |
| Dialysis, % (n/N) | 18.2 (39/214) | 10.3 (3/29) | 1 |
| Tracheostomy, % (n/N) | 8.9 (19/214) | 13.8 (4/29) | 1 |
| VAD, % (n/N) | 35.5 (76/214) | 44.8 (13/29) | 0.412 |
| IMV during hospitalization, % (n/N) | 67.3 (144/214) | 75.9 (22/29) | 0.402 |
| Duration of IMV, median (IQR, n) - d | 9 (6 - 14, n=127) | 9 (5.2 - 16, n=22) | 0.923 |
| IMV at first 24 hours |  |  |  |
| Tidal Volume, median (IQR, n) -ml/kg | 6.1 (6 - 6.8, n=120) | 6.4 (5.8 - 7.4, n=23) | 0.906 |
| Minute Volume, median (IQR, n) -l/min | 11 (9.2 - 12.7, n=124) | 10.5 (9.3 - 12.2, n=21) | 0.753 |
| Compliance, median (IQR, n) - mlcmH_2_O−1 | 30 (23.7 - 40.6, n=113) | 27.3 (23.1 - 28.6, n=17) | 0.078 |
| Respiratory rate, median (IQR, n) - rpm | 30 (26 - 35, n=124) | 31.5 (27.5 - 36.7, n=20) | 0.239 |
| FiO_2,_ median (IQR, n) - % | 50 (40 - 60, n=124) | 50 (50 - 60, n=20) | 0.401 |
| PEEP, median (IQR, n) - cmH_2_O | 10 (8 - 12, n=123) | 10 (10 - 12, n=20) | 0.116 |
| Plateau pressure, median (IQR, n) - cmH_2_O | 22.5 (19 - 26, n=111) | 24 (21.7 - 28.2, n=16) | 0.049 |
| Driving pressure, median (IQR, n) - cmH_2_O | 12 (10 - 15, n=113) | 12.5 (11.7 - 15.2, n=16) | 0.356 |
| PaO_2_/FIO_2,_ median (IQR, n) - % | 155 (120 - 209, n=123) | 138 (108.5 - 188.2, n=20) | 0.537 |
| Tidal Volume ≥ 8, % (n/N) | 3.7 (8/214) | 17.2 (5/29) | 0.037 |
| Respiratory rate ≥ 35, % (n/N) | 0.5 (1/214) | 0 (0/29) | 1 |
| Compliance ≤ 20, % (n/N) | 7.9 (17/214) | 13.8 (4/29) | 0.477 |
| Compliance ≥ 40, % (n/N) | 12.6 (27/214) | 3.4 (1/29) | 0.119 |
| FiO_2_% ≥ 80, % (n/N) | 6.5 (14/214) | 6.9 (2/29) | 1 |
| PEEP ≤ 8, % (n/N) | 19.6 (42/214) | 10.3 (3/29) | 0.079 |
| PEEP ≥ 14, % (n/N) | 4.7 (10/214) | 13.8 (4/29) | 0.125 |
| Plateau pressure ≥ 28, % (n/N) | 6.1 (13/214) | 17.2 (5/29) | 0.065 |
| Driving pressure ≥ 15, % (n/N) | 15 (32/214) | 17.2 (5/29) | 0.577 |
| PaO_2_/FIO_2_ ≤ 150, % (n/N) | 28.5 (61/214) | 37.9 (11/29) | 0.638 |
| PaO_2_/FIO_2_ ≤ 100, % (n/N) | 9.8 (21/214) | 13.8 (4/29) | 0.762 |
| Values are presented as median [IQR, n] or % (n/N) or mean ± SD (n). *Abbreviations:* BMI, body mass index; COPD, chronic obstructive pulmonary disease; CRP, c-reactive protein; d, days; FIO_2_, inspired fraction of oxygen; ICU, intensive care unit. IMV, invasive mechanical ventilation; PaO_2_, arterial partial pressure of oxygen; PEEP, positive end-expiratory pressure; PFTs, pulmonary function tests; SAPS3, Simplified Acute Physiology Score 3; SD, standard deviation; VAD, vasoactive drugs; yr, years. | | | |
