# Supplementary material for: Long-term respiratory follow-up of ICU hospitalized COVID-19 patients: Prospective cohort study
Source: PLoS One. 2023 Jan 20;18(1):e0280567. doi: 10.1371/journal.pone.0280567 (PMC9858876; doi:10.1371/journal.pone.0280567)
Supplement: S5 Table — Values are presented as % (n/N). Abbreviations: COPD, chronic obstructive pulmonary disease; FIO2, inspired fraction of oxygen; IMV, invasive mechanical ventilation; PaO2, arterial partial pressure of oxygen; PEEP, positive end-expiratory pressure. (DOCX) [file pone.0280567.s006.docx]

**Supporting Information S5 Table**

**Long-term respiratory follow-up of ICU hospitalized COVID-19 patients: prospective cohort study**

Carlos Roberto Ribeiro Carvalho1, Celina Almeida Lamas1, Rodrigo Caruso Chate2, João Marcos Salge1, Marcio Valente Yamada Sawamura2, André L. P. de Albuquerque1, Carlos Toufen Junior1, Daniel Mario Lima3, Michelle Louvaes Garcia1, Paula Gobi Scudeller1, Cesar Higa Nomura2, Marco Antonio Gutierrez3, Bruno Guedes Baldi1, HCFMUSP Covid-19 Study Group*

1 Pulmonary Division, Heart Institute (InCor), Hospital das Clínicas, Faculdade de Medicina, Universidade de São Paulo (HCFMUSP), Sao Paulo, SP, Brazil.

2 Radiology Institute (InRad), Hospital das Clínicas, Faculdade de Medicina, Universidade de São Paulo (HCFMUSP), Sao Paulo, SP, Brazil.

3 Informatics Division, Heart Institute (InCor), Hospital das Clínicas, Faculdade de Medicina, Universidade de São Paulo (HCFMUSP), Sao Paulo, SP, Brazil.

*The complete membership of the author group can be found in the Acknowledgments.

**S5 Table.**

| S5 Table. Complementary demographic and clinical characteristics of patients with completed chest CT and lung function results stratified by chest CT lesion severity. | | | |
| --- | --- | --- | --- |
|  | **Without Fibrotic-Like Changes (N=89)** | **With Fibrotic-Like Changes (N=86)** | **p-value** |
| Comorbidities |  |  |  |
| Chronic Kidney Disease, % (n/N) | 7.9 (7/89) | 5.9 (5/84) | 0.767 |
| Diabetes, % (n/N) | 38.2 (34/89) | 41.9 (36/86) | 0.646 |
| COPD, % (n/N) | 12.4 (11/89) | 4.6 (4/86) | 0.310 |
| Hypertension, % (n/N) | 48.3 (43/89) | 65.1 (56/86) | 0.098 |
| Smoke History, % (n/N) | 38.2 (34/89) | 44.2 (38/86) | 0.445 |
| IMV at first 24 hours |  |  |  |
| FiO_2,_ median (IQR, n) - % | 50 (40 - 60, n=50) | 50 (40 - 60, n=61) | 1 |
| Tidal Volume ≥ 8, % (n/N) | 8 (4/50) | 6.9 (4/58) | 1 |
| Respiratory rate ≥ 35, % (n/N) | 0 (0/87) | 1.2 (1/86) | 1 |
| Compliance ≤ 20, % (n/N) | 14.9 (7/47) | 9.4 (5/53) | 0.54 |
| FiO_2_% ≥ 80, % (n/N) | 8 (4/50) | 14.7 (9/61) | 1 |
| PEEP ≤ 8, % (n/N) | 42.9 (21/49) | 24.6 (15/61) | 0.195 |
| PEEP ≥ 14, % (n/N) | 8.2 (4/49) | 8.2 (5/61) | 1 |
| PaO_2_/FIO_2_ ≤ 100, % (n/N) | 20.4 (10/49) | 14.7 (9/61) | 0.457 |
| Values are presented as % (n/N). *Abbreviations:*  COPD, chronic obstructive pulmonary disease; FIO_2_, inspired fraction of oxygen; IMV, invasive mechanical ventilation; PaO_2_, arterial partial pressure of oxygen; PEEP, positive end-expiratory pressure. | | | |
